# Comparing the Feasibility of S-Patch vs 24-Hour Holter in Frail Older People With Falls: Randomized Open-Label Crossover Pilot Study

**DOI:** 10.2196/81761

**Published:** 2026-08-14

**Authors:** Barbara Rosario, Vern Hsen Tan, Vivian Cantiller Barrera, Hnin Tun Mon, Clara Yi-En Seah, Colin Yeo

**Affiliations:** 1 Department of Geriatric Medicine, Changi General Hospital, 2 Simei Street 3, Singapore, 529889, Singapore, 65 84829406; 2 Department of Cardiology, Changi General Hospital, Singapore, Singapore; 3 Health Services Research, Changi General Hospital, Singapore, Singapore; 4 Department of Emergency Medicine, Changi General Hospital, Singapore, Singapore

**Keywords:** falls, frailty, geriatrics, arrhythmia, syncope, monitoring

## Abstract

**Background:**

Falls commonly cause injuries in older adults and can present with syncope or presyncope. Typically, frail older adults with cognitive impairment are underrepresented in research and experience difficulty tolerating conventional ECG monitoring devices. S-Patch is a novel, lightweight dual-lead device designed to provide continuous ECG monitoring for up to 72 hours with minimal interference to daily activities.

**Objective:**

This study aimed primarily to evaluate the feasibility, tolerability, and adherence of the S-Patch compared with standard 24-hour Holter monitoring in older adults (aged ≥60 years) presenting with unwitnessed falls, syncope, or near-syncope, as assessed by wear duration, completion rates, and patient-reported experience. The secondary objective of this study was to assess device usability and compare arrhythmia detection rates between the devices in a real-world setting.

**Methods:**

This prospective, single-center, randomized, open-label, crossover feasibility pilot study was conducted from March 8, 2020, to February 28, 2023. Participants were randomized 1:1 into group A (Holter followed by S-Patch) or group B (S-Patch followed by Holter), enabling up to 4 days of continuous monitoring.

**Results:**

Seventy patients were enrolled, of whom 32 (45.7%) had cognitive impairment, and the majority were frail. Group A participants were older (86.9 vs 82.9 years) with higher use of dementia medications (6/35, 17.1% vs 3/35, 8.6%). Frailty was highly prevalent across the cohort, and fear of falling was significantly greater among frail patients (*P*=.04). Both devices were well tolerated, with no difference in overall device preference, but S-Patch enabled substantially longer monitoring durations compared to Holter. Mean S-Patch wear time exceeded 60 hours and was significantly longer when the device was applied first (68.8 vs 62.9 h; *P*=.04), suggesting acceptance and adherence. Importantly, tolerability was maintained in frail patients with cognitive impairment. Arrhythmia detection rates were observed as comparable, with both devices identifying one clinically significant arrhythmia, resulting in pacemaker implantation.

**Conclusions:**

S-Patch is a feasible, well-tolerated cardiac monitoring device in frail older adults and those with cognitive impairment. Compared with Holter, S-Patch supported longer continuous monitoring without compromising patient acceptability, an important consideration in real-world clinical practice.

## Introduction

Falls are the most common cause of injury in older people, with 10% of falls resulting in serious injuries and injury-related disability [1-3]. One-third of community dwellers over 65 years of age will fall each year, and half of those will fall again [4]. Falls-related injuries are the fifth leading cause of death in older people [5] and one of the most frequent reasons for admission to long-term care institutions in previously independent older people [6].

Long-term monitoring studies suggest that arrhythmias are present in a substantial proportion of patients with unexplained falls, with a subset attributable to treatable rhythm disturbances [2]. However, establishing this relationship is often challenging in clinical practice, particularly in unwitnessed events or when cognitive impairment limits history taking.

Syncope may present as a fall in the absence of a witness account, particularly when coupled with amnesia [2]. First-time syncope is associated with an 80% increased 1-year risk of fall-related injuries, such as fractures and traumatic head injuries, compared with an age- and sex-matched group [7]. Age and multiple comorbidities, including cardiac arrhythmias, are independent risk factors for falls and collectively increase the likelihood of unexplained falls [8,9].

In a large cohort of emergency department (ED) trauma cases (N=5420), 3.3% of falls were attributed to syncope [10], and independent predictors of cardiac syncope were advanced age (>65 y), coronary artery disease, and the presence of pathologic Q waves [10]. Syncope and falls clinic reports identified cardiac causes (rhythm or conduction disorders) in 44% of patients [11], and after identification of an underlying cause, permanent pacemaker (PPM) implantation was associated with a reduced falls risk [12]. Although multifactorial interventions for falls have been less effective in preventing falls-related injuries [13], falls related to arrhythmias remain potentially modifiable factors.

Current guidelines recommend cardiovascular assessment, including ambulatory ECG monitoring, in the evaluation of falls and syncope [14-17]. However, a major limitation in older populations is the tolerability and practicality of monitoring devices. Conventional Holter monitors are relatively bulky, require multiple leads, and impose restrictions on daily activities such as bathing, which may reduce adherence, especially in frail individuals with cognitive impairment. While implantable loop recorders provide prolonged monitoring, their invasive nature limits acceptability in many patients.

As a result, there remains an unmet need for noninvasive, well-tolerated monitoring solutions that can be used effectively in real-world older populations. The S Patch3-Cardio/S-Patch Ex (S-Patch) is a lightweight, novel continuous heart rhythm recording device with a single wire. The S-Patch has been compared with Holter and telemetry [18], and this study aims to compare the tolerability, feasibility, and adherence of the S-Patch with the standard Holter device in an older population. We postulate that it will be as tolerable and efficacious as the Holter device. To our knowledge, this is the first study comparing the S-Patch with the Holter device in an older population in Singapore.

## Methods

### Hypothesis

The hypothesis was that S-Patch is at least as preferred as Holter.

### Primary Objective

The primary objective of this study was to evaluate tolerability and adherence of the S-Patch compared with Holter, assessed by wear duration, completion rates, and patient-reported experience.

### Secondary Objective

The secondary objective of this study was to assess device usability and to compare arrhythmia detection rates between devices in a real-world situation.

### Recruitment, Consent, and Randomization

This prospective, single-center, randomized, open-label, crossover pilot study was performed between March 8, 2020, and February 28, 2023, at a tertiary hospital in Singapore. Eligible patients included older adults aged 60 years and older, attending the ED for an unwitnessed fall, syncope, or presyncope, and referred for Holter testing. Exclusion criteria included patients with known significant bradyarrhythmia, PPM, or inability to tolerate Holter monitoring.

Eligible patients were randomized into 2 groups using a block randomization method with a 1:1 ratio and were randomly allocated to either group A (Holter for 24 h followed sequentially by S-Patch for 72 h) or group B (S-Patch for 72 h followed sequentially by Holter for 24 h), with both monitors worn for up to 4 days. An events diary and a questionnaire were used to evaluate their preference between Holter and the S-Patch recorder. The visit schedule is shown in Multimedia Appendix 1, the user preference questionnaire in Multimedia Appendix 2, and the event diary in Multimedia Appendix 3.

### Device Information, Data Recording, and Analysis

**Figure 1.**
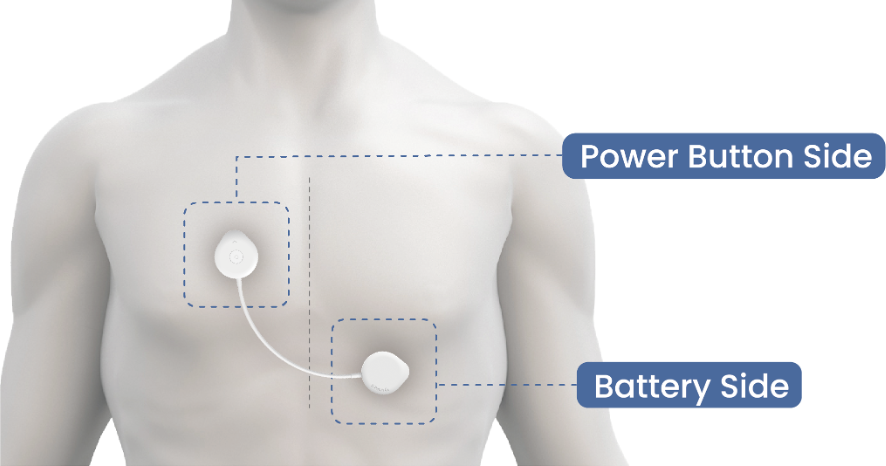
S-Patch electrode placement. Permission was obtained for reproduction of the S-Patch3-Cardio/S-Patch Ex placement image in this manuscript.

The S-Patch is a lightweight device containing a bioprocessor that continuously collects ECG data (for up to 72 h) and transmits them in real time via a Bluetooth connection to an Android smartphone app. The initial coupling requires an internet connection that pairs the device with a precreated patient profile. The device is easily applied with only 2 standard ECG electrodes placed typically in the V2-V3 position (Figure 1). The S-Patch sends data to the phone (to be kept within 3 m) via Bluetooth continuously and does not require an internet connection. The app allows the patient to be able to add symptoms or activities either by using a precreated list or by entering their own information. At the end of the test period, the recorded information is uploaded to a cloud-based database via an internet connection and is analyzed by an algorithm, after which a report is generated and reviewed by a consultant cardiologist according to the following clinical criteria for the definition of a major abnormality:

1. Ventricular tachycardia of 3 or more consecutive premature ventricular beats
2. Sinus or ventricular pauses of 3 or more seconds
3. Bradycardia with a heart rate of less than 30 beats per minute
4. Mobitz type II atrioventricular block
5. Complete heart block
6. The definition or clinical criteria for minor abnormalities include the following:
  - Multiple ventricular ectopic beats at a rate of 100 or more premature beats per hour
  - Paroxysmal supraventricular tachycardia for 100 or more beats per hour
  - Bradycardia with a heart rate of 30 to 39 beats per minute
  - Mobitz type I atrioventricular block

Results from the Holter were reviewed and analyzed as per current clinical practice. The data comparison recorded is shown in Multimedia Appendix 4.

### Data Collection, Power Calculation, and Statistical Analysis

Data collection included demographic data on age, sex, race, BMI, and comorbidities, including an existing or new diagnosis of dementia or mild cognitive impairment. Cognitive screening was undertaken using the Abbreviated Mental Test (AMT), with a score of ≥7 considered normal, and functional status was assessed using the Barthel score [19] and categorized into independent (20), mild (16-19), moderate (11-15), and severe disability (≤10). The Short Physical Performance Battery (SPPB) assessment [20] was conducted, with scores ranging from 0 (worst performance) to 12 points (best performance). Falls and fear of falling were ascertained by screening questions, “Do you have fear of falling?” and “How many falls have you sustained in the last year?” Frailty was assessed using the Clinical Frailty Scale (CFS) [21], a widely used judgment-based frailty screening tool and nationally agreed community frailty screening tool [22]. Outcomes derived from hospitalization data included length of stay (LOS); ED reattendance at 7 days, 30 days, or 6 months; emergency hospital readmission at 30 days or 6 months; and inpatient mortality.

Power calculation determined that 116 patients were required to achieve 80% power to detect a difference in abnormality rates of 20%, assuming a significance level of 5%. Assuming a 20% dropout rate, the study aimed to recruit 140 patients.

Participant characteristics at baseline and secondary outcomes were examined according to their statistical distributions. Parametric continuous data were compared using 2-tailed *t* tests and are presented as mean (SD), while nonparametric data were analyzed with Mann-Whitney *U* tests and are presented as median (IQR). Categorical data comparing participant characteristics and assigned groups were evaluated using chi-square tests or Fisher exact tests and are presented as proportions. The significance level was set at α=.05, and all tests were 2-tailed. Statistical analysis was performed using Stata statistical software (version 16; StataCorp).

### Ethical Considerations

The study protocol was approved by the SingHealth Centralized Institutional Review Board (CIRB; ethics approval number 2019/2969) and Clinical Trial Registration 2019/2960. Informed consent was obtained from all participants, and if patients lacked capacity to make decisions, informed consent was obtained from their legally appointed representatives.

## Results

### Screening and Recruitment

**Figure 2.**
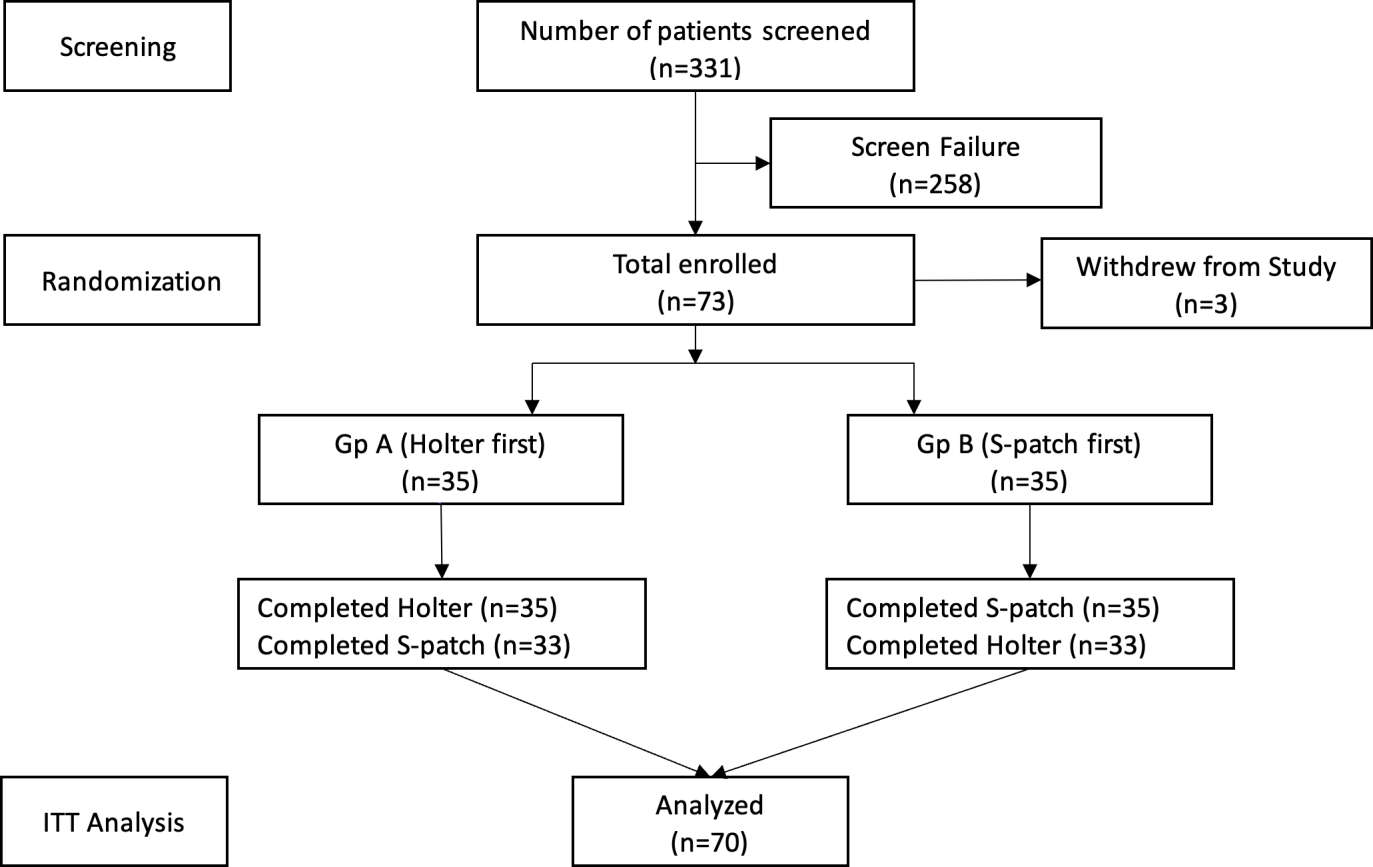
figure Screening and recruitment. Gp A: group A; Gp B: group B; ITT: intention-to-treat.

The study recruited 73 patients, 3 of whom withdrew from the study, and 70 patients completed the study interventions. Group A received Holter first, followed immediately by S-Patch, and Group B received S-Patch first, followed immediately by Holter, providing up to 4 days of continuous monitoring. Two patients received only Holter (one declined S-Patch and the other had no recording of S-Patch as data were not transferred from the Android smartphone app). Two patients refused Holter monitoring and only had S-Patch monitoring. There were 35 patients in both groups, of whom 33 in both groups completed sequential Holter and S-Patch monitoring or vice versa. The majority of patients completed cardiac monitoring during their inpatient stay. Screening and recruitment are shown in Figure 2.

### Demographics

Group A participants were older than Group B participants (mean age 86.9, SD 5.8 y vs 82.9, SD 7.1 y), with a similar proportion of females in both groups. There were more Chinese patients (30/35, 85.7% vs 22/35, 62.9%) and more Indian patients (4/35, 11.4% vs 2/35, 5.7%) in Group A and more Malay patients in Group B (0% vs 8/35, 22.9%; Table 1). BMI was similar in both groups, as was the prevalence of hypertension (29/35, 82.9% vs 30/35, 85.7%) and diabetes mellitus (17/35, 48.6% vs 19/35, 54.3%), but Group B had more patients with atrial fibrillation (AF; 5/35, 14.3% vs 10/35, 28.6%) and stroke (8/35, 22.9% vs 14/35, 40%), although there were no statistical differences in comorbidities between the 2 groups. Few patients were active smokers across the groups (2/35, 5.7% vs 2/35, 5.7%). Only a small proportion of patients (1/35, 2.9% vs 2/35, 5.7%) had left ventricular ejection fraction below 40%. There were no differences in the use of antiarrhythmic or dementia medications (6/35, 17.1% vs 3/35, 8.6%; Table 1).

**Table 1.** Demographics, comorbidities, cognitive impairment, frailty and functional assesssments, device tolerance, duration and arrhythmia detection, and hospitalization usage.

Characteristic	Group A (Holter first), n=35	Group B (S-Patch first), n=35	*P* value
Gender, n (%)			>.99
Female	22 (62.9)	21 (60)	
Male	13 (37.1)	14 (40)	
Race, n (%)			.005
Chinese	30 (85.7)	22 (62.9)	
Malay	0 (0)	8 (22.9)	
Indian	4 (11.4)	2 (5.7)	
Others	1 (2.9)	3 (8.6)	
Age (y), mean (SD)	86.9 (5.8)	82.9 (7.1)	.01
BMI (34 vs 34), mean (SD)	22.1 (4.0)	22.9 (4.5)	.41
Comorbidities, n (%)			
Diabetes mellitus	17 (48.6)	19 (54.3)	.81
Hypertension	29 (82.9)	30 (85.7)	>.99
CCF^a^	2 (5.7)	0 (0)	.49
Stroke/TIA^b^	8 (22.9)	14 (40)	.20
AF^c^	5 (14.3)	10 (28.6)	.24
IHD^d^	9 (25.7)	11 (31.4)	.79
COPD^e^	0 (0)	1 (2.9)	>.99
Parkinson disease	4 (11.4)	4 (11.4)	>.99
Smoker, n (%)	2 (5.7)	2 (5.7)	>.99
LVEF^f^ (%; 30 vs 28), mean (SD)	57.8 (7.3)	56.8 (7.3)	.58
LVEF <40, n (%)			>.99
No	34 (97.1)	33 (94.3)	
Yes	1 (2.9)	2 (5.7)	
Medications, n (%)			
None	8 (22.9)	15 (42.9)	.13
Beta blocker	13 (37.1)	15 (42.9)	.81
Calcium channel blocker	16 (45.7)	10 (28.6)	.22
Digoxin	1 (2.9)	1 (2.9)	>.99
Dementia (donepezil and memantine)	6 (17.1)	3 (8.6)	.48
Cognition, n (%)			
Dementia			.47
No	17 (48.6)	21 (60)	
Yes	18 (51.4)	14 (40)	
Abbreviated Mental Test (AMT; 28 vs 29), mean (SD)	6.5 (3.0)	6.5 (3.3)	.98
Rockwood Clinical Frailty Score (CFS), mean (SD)	4.8 (1.3)	4.5 (1.5)	.31
CFS groups, n (%)			.81
Not frail/robust (1-3)	6 (17.1)	7 (20)	
Mildly frail (4-5)	17 (48.6)	19 (54.3)	
Moderate/severe frail (6-9)	12 (34.3)	9 (25.7)	
Barthel Index (35 vs 35), mean (SD)	15.8 (4.6)	16.5 (4.4)	.54
Barthel Index subgroups, n (%)			.37
Independent (20)	9 (25.7)	14 (40)	
Mild disability (16-19)	13 (37.1)	10 (28.6)	
Moderate disability (11-15)	6 (17.1)	8 (22.9)	
Severe disability (≤10)	7 (20)	3 (8.6)	
Short Physical Performance Battery Test (SPPB; 24 vs 25), mean (SD)	3.7 (2.8)	3.9 (3.0)	.84
Fear of falling, n (%)			.21
No	18 (60)	14 (42.4)	
Yes	12 (40)	19 (57.6)	
Number of falls in the past 12 mo, mean (SD)	2.2 (1.9)	2.0 (1.9)	.67
Duration of device (h), mean (SD)			
Holter (35 vs 33)	22.6 (1.3)	22.5 (1.1)	.52
S-Patch (33 vs 35)	62.9 (13.9)	68.8 (9.5)	.04
S-Patch analysis time (33 vs 35; h)	53.1 (13.4)	58.9 (13.6)	.08
Symptoms, n (%)			.72
Unwitnessed fall	21 (60)	17 (48.6)	
Syncope	5 (14.3)	6 (17.1)	
Near syncope	0 (0)	0 (0)	
NA	9 (25.7)	12 (34.3)	
Arrhythmia events by device^g^, n (%)			
Holter (Yes)	10 (28.6)	15 (42.9)	.32
S-Patch (Yes)	9 (25.7)	9 (25.7)	>.99
Holter: sinus node disease, n (%)			>.99
No	31 (88.6)	29 (87.9)	
Yes	4 (11.4)	4 (12.1)	
Holter: AV^h^ node disease			>.99
No	32 (91.4)	31 (93.9)	
Yes	3 (8.6)	2 (6.1)	
Holter: Tachyarrhythmia, n (%)			.13
None	31 (88.6)	23 (69.7)	
SVT^i^	0 (0)	1 (3)	
AF/Flutter	4 (11.4)	8 (24.3)	
VT^j^/VF^k^	0 (0)	1 (3)	
S-Patch: sinus node disease, n (%)			—^l^
No	33 (100)	35 (100)	
Yes	0 (0)	0 (0)	
S-Patch: AV node disease, n (%)			.67
No	30 (90.9)	33 (94.3)	
Yes	3 (9.1)	2 (5.7)	
S-Patch: Tachyarrhythmia, n (%)			.41
None	27 (81.8)	28 (80)	
SVT	3 (9.1)	1 (2.9)	
AF/Flutter	3 (9.1)	6 (17.1)	
VT/VF	0 (0)	0 (0)	
Preference (Holter vs S-Patch), n (%)			.36
Holter	3 (8.8)	7 (21.2)	
S-Patch	22 (64.7)	17 (51.5)	
No preference	9 (26.5)	9 (27.3)	
None of the device	0 (0)	0 (0)	
Prefer Holter, n (%)			
User friendly and ease of operation	2 (5.7)	3 (8.6)	>.99
Comfortable	1 (2.9)	0 (0)	>.99
Does not affect daily activities	1 (2.9)	3 (8.6)	.61
Others, please specify	2 (5.7)	2 (5.7)	>.99
Prefer S-Patch, n (%)			
User friendly and ease of operation	13 (37.1)	9 (25.7)	.44
Comfortable	16 (45.7)	12 (34.3)	.46
Does not affect daily activities	11 (31.4)	7 (20)	.41
Others, please specify	1 (2.9)	4 (11.4)	.36
No preference, n (%)			
User friendly and ease of operation	3 (8.6)	2 (5.7)	>.99
Comfortable	4 (11.4)	8 (22.9)	.34
Does not affect daily activities	4 (11.4)	4 (11.4)	>.99
Others, please specify	1 (2.9)	0 (0)	>.99
Neither device, n (%)			—
User friendly and ease of operation	0 (0)	0 (0)	
Comfortable	0 (0)	0 (0)	
Does not affect daily activities	0 (0)	0 (0)	
Others, please specify	0 (0)	0 (0)	
Holter satisfaction, n (%)			>.99
Extremely satisfied	0 (0)	0 (0)	
Very satisfied	2 (66.7)	4 (57.1)	
Moderately satisfied	1 (33.3)	3 (42.9)	
Not so satisfied	0 (0)	0 (0)	
Dissatisfied	0 (0)	0 (0)	
S-Patch satisfaction, n (%)			.07
Extremely satisfied	6 (27.3)	1 (5.9)	
Very satisfied	14 (63.6)	10 (58.9)	
Moderately satisfied	2 (9.1)	6 (35.3)	
Not so satisfied	0 (0)	0 (0)	
Dissatisfied	0 (0)	0 (0)	
Hospitalization usage			
Hospital admission at index visit, n (%)			>.99
No	2 (5.7)	3 (8.6)	
Yes	33 (94.3)	32 (91.4)	
Total number of hospitalization days (34 vs 35), mean (SD)	0.68 (0.91)	0.77 (1.48)	.75
Length of stay (days; 33 vs 32), mean (SD)	17.9 (16.2)	16.8 (20.8)	.80
ED^m^ reattendance (6 mo), n (%)			
Yes	13 (37.1)	7 (20)	.19
Hospital readmission (6 mo), n (%)			
Yes	12 (34.3)	4 (11.4)	.04
Mortality, n (%)	0 (0)	1 (2.9)	>.99

a CCF: congestive cardiac failure.

b TIA: transient ischemic attack.

c AF: atrial fibrillation.

d IHD: ischemic heart disease.

e COPD: chronic obstructive pulmonary disease.

f LVEF: left ventricular ejection fraction.

g Arrhythmia events relate to events detected in individual patients but patients may have more than one reported arrhythmia event.

h AV: atrioventricular.

i SVT: supraventricular tachycardia.

j VT: ventricular tachycardia.

k VF: ventricular fibrillation.

l Not applicable.

m ED: emergency department.

### Tolerability of S-Patch vs Holter

Holter device was tolerated well in both groups and was worn for similar periods in group A vs group B (mean 22.6, SD 1.3 h vs 22.5, SD 1.1 h; *P*=.52; Table 1). S-Patch was also tolerated well in both groups; however, the device was worn for longer in group B, where S-Patch was administered first (mean 62.9, SD 13.4 h vs 68.8, SD 9.5 h*; P*=.04; Table 1). The overall S-Patch analysis time was longer in group B (mean 53.1, SD 13.4 h vs 58.9, SD 13.6 h; *P*=.08) but did not reach statistical significance. Although there was no difference in the patient’s device preference between groups, a higher proportion of participants in group A (3 vs 22) vs group B (7 vs 17) preferred S-Patch, with 9 participants in both groups having no preference (Table 1). Compliance with completion of preference questionnaires was high in both groups (group A, 34/35, 97% and group B, 33/35, 94%; Table 1).

### Cognition and Dementia

Overall, 45.7% (32/70) of patients had dementia or cognitive impairment, either known or newly diagnosed during the index admission, with similar numbers of patients with cognitive impairment across both groups (group A: 18/35, 51.4% vs group B: 14/35, 40%; Table 1). Only a small proportion of patients (9/70, 12.9%) were taking anticholinesterase inhibitors (ACHi; group A: 6/35, 17.1% vs group B: 3/35, 8.6%). None of the patients on ACHi had symptomatic bradyarrhythmia. Average AMT score was 6.5 for both groups (Table 1). In terms of the duration of monitoring undertaken in those with or without cognitive impairment, both devices were tolerated well, and Holter was worn for 22.5 (SD 1.4) hours vs 22.6 (SD 1.1 hours; *P*=.66) and S-Patch for 67.5 (SD 9.5) hours vs 64.6 (SD 11.3 hours; *P*=.33; Table 2).

**Table 2.** Cognitive impairment and dementia status (N=70).

Characteristic	No known dementia, n=38 (54.3%)	Yes dementia, n=32 (45.7%)	*P* value
Barthel Index group, n (%)			.001
Independent (20)	17 (44.7)	6 (18.8)	
Mild disability (16-19)	16 (42.2)	7 (21.9)	
Moderate disability (11-15)	4 (10.5)	10 (31.3)	
Severe disability (≤10)	1 (2.6)	9 (28.0)	
CFS^a^ groups, n (%)			.001
Not frail/robust (1-3)	13 (34.2)	0 (0)	
Mildly frail (4-5)	23 (60.5)	13 (40.6)	
Moderate/severe frail (6-9)	2 (5.3)	19 (59.4)	
AMT^b^, n (%)			<.001
Score (≥7)	26 (68.4)	7 (21.9)	
Score (0‐6)	5 (13.2)	19 (59.4)	
Not done	7 (18.4)	6 (18.8)	
Duration of device (h), mean (SD)			
Holter	22.6 (1.1)	22.5 (1.4)	.66
S-Patch	64.6 (13.8)	67.5 (9.5)	.33
S-Patch analysis time (h)	57.8 (14.6)	54.0 (12.4)	.26
Fear of falling, n (%)			.13
No	21 (60)	11 (39.3)	
Yes	14 (40)	17 (60.7)	
Unable to answer	0 (0)	0 (0)	
Number of falls in the past 12 mo, mean (SD)	1.9 (1.8)	2.4 (2.0)	.28
ED attendance, n (%)			—
No	0 (0)	0 (0)	
Yes	37 (100)	32 (100)	
Total number of hospitalization days, mean (SD)	0.49 (0.9)	1.0 (1.5)	.08
Length of stay (d), mean (SD)	13.2 (9.4)	21.7 (24.0)	.07
Mortality, n (%)	0 (0)	1 (3.1)	.46
Hospital readmit (6 mo), n (%)			
Yes	6 (15.8)	10 (31.3)	.16
ED readmit (6 mo), n (%)			
Yes	9 (23.7)	11 (34.4)	.43

a CFS: Clinical Frailty Scale.

b AMT: Abbreviated Mental Test.

### Frailty, Falls, and Functional Status

Frailty assessed using CFS was similar between group A and group B, with 34.3% (12/35) vs 25.7% (9/35) of participants being moderately or severely frail, 48.6% (17/35) vs 54.3% (19/35) being very mildly or mildly frail, and 17.1% (6/35) vs 20% (7/35) not being frail. There were more patients with cognitive impairment in the moderate or severely frail groups (19/35, 59.4% vs 0/35, 0%) compared to nonfrail (*P*=.001; Table 2). There was a trend toward more falls with increasing frailty (5/13, 38.5% in CFS 1‐3 vs 19/36, 52.8% in CFS 4 and 5 vs 14/21, 66.7% in CFS 6‐9), which did not reach statistical significance (Table 3). However, fear of falling was significantly higher in frail patients (4/13, 30.8% in CFS 1‐3 vs 14/32, 43.8% in CFS 4 and 5 vs 13/18, 72.2% in CFS 6‐9; *P*=.04; Table 3) but did not translate to increased hospital usage.

**Table 3.** Barthel status and the impact of function on tolerance of the device, fear of falls, and hospital usage.

Characteristics	Independent (20), n=23 (32.9%)	Mild disability (16-19), n=23 (32.9%)	Moderate disability (11-15), n=14 (24.3%)	Severe disability (≤10), n=10 (14.3%)	*P* value
CFS^a^ groups, n (%)					.001
Not frail/robust (1-3)	10 (43.5)	3 (13)	0 (0)	0 (0)	
Mildly frail (4-5)	13 (56.5)	19 (82.6)	4 (28.6)	0 (0)	
Moderate/severe frail (6-9)	0 (0)	1 (4.4)	10 (71.4)	10 (100)	
AMT^b^ groups, n (%)					.05
Normal (≥7)	12 (52.2)	14 (60.9)	6 (42.9)	1 (10)	
Cognitive impairment (0‐6)	5 (21.7)	8 (34.8)	5 (35.7)	6 (60)	
Not done	6 (26.1)	1 (4.4)	3 (21.4)	3 (30)	
Dementia, n (%)					.001
No	17 (73.9)	16 (69.6)	4 (28.6)	1 (10)	
Yes	6 (26.1)	7 (30.4)	10 (71.4)	9 (90)	
Duration of device (h), mean (SD)					
Holter	22.9 (1.3)	22.4 (1.2)	22.2 (1.1)	22.8 (1.2)	.30
S-Patch	64.6 (14.2)	65.8 (12.0)	70.8 (4.3)	62.9 (13.9)	.39
S-Patch analysis time (h)	56.8 (14.1)	55.9 (13.8)	58.2 (12.2)	52.3 (15.5)	.78
Fear of falling, n (%)					.40
No	13 (65)	11 (50)	5 (41.7)	3 (33.3)	
Yes	7 (35)	11 (50)	7 (58.3)	6 (66.7)	
Unable to answer	0 (0)	0 (0)	0 (0)	0 (0)	
Number of falls in the past 12 mo, mean (SD)	1.8 (1.4)	1.7 (1.5)	3.4 (2.9)	1.7 (1.3)	.03
ED^c^ attendance, n (%)					.96
No	0 (0)	0 (0)	0 (0)	0 (0)	
Yes	22 (100)	21 (100)	17 (100)	9 (100)	
Total number of hospitalization days, mean (SD)	0.6 (0.9)	0.4 (0.5)	1.1 (2.0)	1.1 (1.4)	.22
Length of stay (d), mean (SD)	17.1 (22.8)	13.5 (10.4)	26.6 (23.5)	13.9 (9.2)	.21
Mortality, n (%)	0 (0)	0 (0)	1 (7.1)	0 (0)	.35
Hospital readmit (6 mo), n (%)					
Yes	7 (30.4)	5 (21.7)	0 (0)	4 (40)	.06
ED readmit (6 mo), n (%)					
Yes	10 (43.5)	5 (21.7)	0 (0)	5 (50)	.007

a CFS: Clinical Frailty Scale.

b AMT: Abbreviated Mental Test.

c ED: emergency department.

Both groups presented predominantly with an unwitnessed fall (21/35, 60% vs 17/35, 48.6%) and fewer with syncope (5/35, 14.3% vs 6/35, 17.1%) or near syncope (9/35, 25.7% vs 12/35, 34.3%). Falls in the preceding 12 months were similar in both group A and group B (2.2 vs 2.0, SD 1.9), with fear of falling also being similar (12/30, 40% vs 19/33, 57.6%). The SPPB was performed in 68% (24/35) of group A and 71% (25/35) of group B participants, with a mean score of 3.7 (SD 2.8) vs 3.9 (SD 3.0; *P*=.84; Table 1), and functional impairment was mostly cited as the reason why patients were unable to perform the SPPB. Fall frequency in the preceding 12 months was higher in those with moderate disability (3.4 falls, SD 2.9) compared with those with severe disability (1.7 falls, SD 1.3), mild disability (1.7 falls, SD 1.5), and independent patients (1.8 falls, SD 1.4; *P*=.03; Table 3).

### Observed Arrhythmia Detection

In terms of arrhythmia events in group A vs group B, Holter identified 28.6% (10/35) vs 42.9% (15/35; *P*=.32) and S-Patch identified 9 vs 9 (25/70, 35.7% vs 18/70, 25.7%; *P*>.99; Table 1). There were no statistically significant differences in sinus node disease, AV nodal disease, or tachyarrhythmia between the group A and group B participants for Holter and S-Patch (Table 1). Outcome comparison between the 2 treatment arms using McNemar test showed no differences in outcome events between Holter and S-Patch monitoring, 25 out of 70 (35.7%) vs 18 out of 70 (25.7%; *P*=.21; Table 4). For agreement analysis using paired data from the crossover design (n=70), a 2 × 2 contingency table (Table 4) was used to compare the detection of clinically significant arrhythmias by both devices. McNemar test showed no significant difference in arrhythmia detection between the 2 devices (*χ*²_1_=2.13, *P*=.21), and Cohen κ was 0.24 (*P*=.02), indicating fair agreement between devices. Using Holter as the reference standard, S-Patch sensitivity was 40% (10/25) and specificity was 82.2% (37/45), giving a positive predictive value of 55.6% (10/18) and a negative predictive value of 71.2% (37/52).

**Table 4.** Outcome comparison between 2 treatment arms and contingency table comparing detection of clinically significant arrhythmias for both devices.^a^

Contingency table	Holter: yes	Holter: no	Total
S-Patch: Yes	10	8	18
S-Patch: No	15	37	52

a McNemar test was used to compare detection of clinically significant arrhythmias between devices. Clinically significant arrhythmias included atrial fibrillation, atrial tachycardia, tachyarrhythmias, and bradyarrhythmias. S-Patch identified clinically significant arrhythmias in 25.7% (18/70) of participants and Holter monitoring identified clinically significant arrhythmias in 35.7% (25/70) of participants (*P*=.21).

S-Patch identified one symptomatic arrhythmia and a pause of >3 seconds, and Holter confirmed sinus arrest, with a long pause suggestive of sinus node disease for which a PPM was recommended. Another patient had an asymptomatic 2.8-second pause on S-Patch, which was not classified as an event.

There was a high level of agreement (κ=0.8584) between the 2 blinded raters (cardiologists) for AF, which was significantly greater than expected by chance (*P*<.001). The observed agreement for artifact was 37.54% and fell slightly below the expected agreement of 41.18%, resulting in a low κ coefficient of 0.0582, but this was not statistically significant (*P*=.21).

### Hospital Usage and Mortality

ED attendance resulted in most patients being admitted to hospital (94.1% vs 91.4%; *P*>.99). Hospital LOS was similar in both groups (mean 17.9, SD 16.2 vs 16.8, SD 20.8; *P*=.80; Table 1). There was only 1 (2.9%, *P*>.99) mortality in group B during the study period (Table 1). Odds ratios for ED reattendance at 7 days, 30 days, or 6 months and for hospital readmission at 30 days or 6 months were similar between the 2 groups (Table 5).

**Table 5.** Comparison of hospital readmission and emergency department reattendance across study groups.

Outcome	Odds ratio or IRR^a^ (95% CI)	*P* value
Hospital readmission^b^ (OR^c^)		
Tx B (S-Patch first)	0.33 (0.11-1.03)	.06
Hospital readmission^b^ (30 d; IRR)		
Tx B (S-Patch first)	0.36 (0.12-1.14)	.08
Hospital readmission^b^ (6 mo; IRR)		
Tx B (S-Patch first)	0.38 (0.13-1.12)	.08
ED^d^ reattendance^b^ (OR)		
Tx B (S-Patch first)	0.42 (0.14-1.24)	.12
ED reattendance^b^ (7 d; IRR)		
Tx B (S-Patch first)	0.25 (0.02-2.76)	.26
ED reattendance^b^ (30 d; IRR)		
Tx B (S-Patch first)	0.50 (0.18-1.41)	.19
ED reattendance^b^ (6 mo) (IRR)		
Tx B (S-Patch first)	0.53 (0.22-1.27)	.15

a IRR: incidence rate ratio.

b Reference: Tx A (Holter first).

c OR: odds ratio.

d ED: emergency department.

ED reattendance within 6 months was high (5/10, 50%) in those whose Barthel score indicated severe disability, 0% (0/14) in those with moderate disability, 21.7% (5/23) in those with mild disability, and 43.5% (10/23) for independent patients (*P*=.007; Table 4). Those with moderate functional disability had a longer average LOS (26.6, SD 23.5 days) compared with those with severe disability (13.9, SD 9.2 days), mild disability (13.5, SD 10.4 days), and independent patients (17.1, SD 22.8 days; *P*=.21; Table 6).

**Table 6.** Clinical Frailty Scale.

CFS^a^	Not frail/robust (1-3), n=13 (18.6%)	Mildly frail (4-5), n=36 (51.4%)	Moderate/severe frail (6-9), n=21 (30.0%)	*P* value
Bathel Index group, n (%)				.001
Independent (20)	10 (76.9)	13 (36.1)	0 (0)	
Mild disability (16-19)	3 (23.1)	19 (52.8)	1 (4.8)	
Moderate disability (11-15)	0 (0)	4 (11.1)	10 (47.6)	
Severe disability (≤10)	0 (0)	0 (0)	10 (47.6)	
AMT^b^ groups, n (%)				.001
Normal (≥7)	10 (76.9)	19 (52.8)	4 (19.1)	
Cognitive impairment (0‐6)	0 (0)	13 (36.1)	11 (52.4)	
Not done	3 (23.1)	4 (11.1)	6 (28.6)	
Dementia, n (%)				.001
No	13 (100)	23 (63.9)	2 (9.5)	
Yes	0 (0)	13 (36.1)	19 (59.4)	
Duration of device (h), mean (SD)				
Holter	23.1 (0.8)	22.3 (1.3)	22.7 (1.2)	.10
S-Patch	61.3 (17.3)	66.9 (10.7)	66.9 (10.6)	.35
S-Patch analysis time (h)	56.6 (16.6)	56.7 (12.8)	54.8 (13.9)	.87
Fear of falling, n (%)				.04
No	9 (69.2)	18 (56.3)	5 (27.8)	
Yes	4 (30.8)	14 (43.8)	13 (72.2)	
Number of falls in the past 12 mo, mean (SD)	1.5 (1.0)	2.0 (1.9)	2.6 (2.2)	.26
ED^c^ attendance, n (%)				>.99
No	0 (0)	0 (0)	0 (0)	
Yes	12 (100)	36 (100)	21 (100)	
Total number of hospitalization days, mean (SD)	0.4 (0.9)	0.6 (0.9)	1.0 (1.7)	.31
Length of stay (d), mean (SD)	9.6 (7.0)	17.9 (19.9)	21.2 (20.0)	.22
Mortality, n (%)	0 (0)	0 (0)	1 (4.8)	.48
Hospital readmit (6 mo), n (%)				>.99
Yes	3 (23.1)	8 (22.2)	5 (23.8)	
ED readmit (6 mo), n (%)				>.99
Yes	4 (30.8)	10 (27.8)	6 (28.6)	

a CFS: Clinical Frailty Scale.

b AMT: Abbreviated Mental Test.

c ED: emergency department.

## Discussion

To our knowledge, this prospective single-center randomized open-label crossover pilot study is the largest to evaluate the tolerability and real-world use of the S-Patch in an older population, with a high prevalence of cognitive impairment and frailty. In this cohort, the S-Patch was well tolerated and enabled longer continuous monitoring durations compared with standard Holter monitoring. These findings address a key gap in clinical practice, where the success of ambulatory ECG monitoring is often limited not by diagnostic capability but by patient adherence and device acceptability.

Compliance with monitoring was good for both devices, but S-Patch was tolerated for longer periods when S-Patch was administered first (group A [Holter first], 62.9, SD 13.4 h vs group B [S-Patch first] 68.8, SD 9.5 h; *P*=.04). In terms of durability, S-Patch also reports the available analysis time, which allows some interpretation of efficacy. Analysis time was 53.1 (SD 13.4) hours vs 58.9 (SD 13.6) hours. The difference between duration worn and available analysis time may be explained by loss of signal or due to interference during patient activities. The requirement for a paired mobile device within close proximity may have introduced practical challenges and may have contributed to reduced data capture in some patients.

This study is unique in its high proportion of vulnerable patients, including those with dementia (32/70, 45.7%) and frailty (57/70, 81.4%). Conventional monitoring approaches are frequently challenging in these groups due to device complexity, discomfort, and interference with daily activities. S-Patch offers an alternative to Holter monitoring, and its lightweight, minimally intrusive design likely contributed to sustained wear time. Importantly, tolerability was maintained despite the need for caregiver support in some cases.

Holter is considered the gold standard for short-term monitoring, and the diagnostic yield of clinically significant arrhythmias varies between 5.2% and 11% [23-25], with older age increasing the likelihood of significant arrhythmia identification [23,25]. Oversensing was more frequent with the S-Patch (75.76%) and, less so, undersensing (6%), which highlights the major challenges of analyzing complex and variable ECG signals. Other studies using S-Patch 2 have shown a cardiologist-level accuracy of 99.8% [26]. In terms of diagnostic performance, arrhythmia detection rates were comparable between devices, with one clinically significant arrhythmia identified by both modalities leading to pacemaker implantation. Although the S-Patch demonstrated lower sensitivity but reasonable specificity relative to Holter, the clinical implications of this finding should be interpreted cautiously. In older adults with falls, short-term monitoring has inherent limitations. Arrhythmias are often intermittent and may not coincide with the monitoring window, and detection in the absence of symptom correlation may not alter management. Conversely, a negative result does not exclude an arrhythmic cause.

In the Multi-Ethnic Study of Atherosclerosis, AF was first detected on days 3 to 14 of monitoring [27], highlighting that extending monitoring to 72 hours may still be insufficient for arrhythmia detection. The infrequent nature of syncopal events makes their relationship to falls challenging, but longer periods of ILR monitoring have shown that 71% of patients with unexplained falls had an underlying arrhythmia that was not initially present but was detected within 9 months using ILR [2]. However, their invasive nature limits widespread acceptability, reinforcing the importance of maximizing tolerability in noninvasive monitoring approaches.

Conversely, ambulatory recording in those with a history of recurrent falls [28] showed no causative arrhythmias and concluded that most arrhythmias detected did not correlate with symptoms [28]. The impact of falls is potentially devastating, and first-time syncope is associated with an 80% increased 1-year risk of fall-related injuries compared with an age- and sex-matched group [7]. AF is also associated with falls in older adults [9], and the prevalence of AF in this study was high at 16.5%, of whom 41% had paroxysmal AF, making detection more challenging [9]. In addition, even those with a history of AF have a 2.5 times greater risk of nonaccidental falls [29], and independent predictors of falls included AF, neurological disorders, and age ≤81 years [29].

Falls remain a multifactorial geriatric syndrome with significant negative consequences, including fear of falling, functional deterioration, anxiety, and depression [7], and older patients feel stigmatized by falls as a marker of aging [30]. This study found that those with moderate functional impairment had increased falls in the preceding 12 months (mean 3.4, SD 2.9; *P*=.03), and frail patients had a greater fear of falling compared with nonfrail patients (13/18, 72.2% vs 4/13, 30.8%; *P*=.04). Cardiac arrhythmias represent one of the few potentially reversible contributors to falls, and improving access to practical and acceptable monitoring strategies is clinically important.

Targeting higher-risk patients, such as those with unwitnessed falls, syncope, or established cardiovascular disease, may further improve diagnostic efficiency [8]. Independent predictors of cardiac syncope include age older than 65 years, the presence of coronary artery disease, and Q waves on the ECG [10]. ECG abnormalities were reported in 64% of syncope clinic attendees, where 26% of ECGs suggested an underlying cardiac cause [11], and high-risk ECG features or elevated cardiac enzymes can guide clinicians in the selection for onward referral or longer-term monitoring [31]. High-risk patients include those post-TAVI, with a high rate of arrhythmia detection, with new-onset AF in 19.6%, and recurrent episodes of AF were detected in those with a history of AF [32].

There were challenges and limitations within this study, which included that this was a single-site, open-label study with a crossover design to provide a comparison for tolerability, and blinding was not possible due to physical differences in the devices. Devices were placed by technicians, and analysis was undertaken by cardiologists not involved in the direct care of the patients to reduce the risk of bias. The randomized crossover design mitigated the risk of bias, and the 2 groups were well balanced. The study was underpowered as only 73 patients from an intended 116 were recruited, which limits the statistical power and increases the likelihood of type II errors. Recruitment of older patients was challenging, consistent with other literature [33]. Reasons for declining the study have been categorized into 4 groups. First, patient-related factors, such as older patients’ reticence to participate in clinical trials, concern about tolerating a device (particularly in those with dementia), and fear of prolonging hospitalization, despite both devices being available for home recordings. Second, consent-related issues, which included the inability or lack of relatives to attend the hospital to provide legally appointed representative consent, particularly during the pandemic restrictions. Third, physician-related factors, in that Holter monitoring was already initiated or the physician preferred monitoring for a longer period. Finally, the COVID-19 pandemic meant that research activities were suspended for long periods, and there was an upgrade and replacement of the S-Patch during which recruitment could not take place. Finally, the majority of Holter and S-Patch recordings were undertaken during hospitalization, as most patients declined home monitoring, and this was the commonest reason cited for withdrawing from the crossover part of the study. Monitoring for arrhythmia during hospitalization may not be reflective or representative of arrhythmias during normal day-to-day activity, which may explain some of the variability in the identification of arrhythmias between the 2 devices.

The sequential design (Holter followed by S-Patch, or vice versa) may also introduce a major confounding factor related to the temporal variability of arrhythmias and makes it challenging to decide if this is due to device failure or the absence of an arrhythmia during the specific monitoring window. This limitation makes a direct comparison of diagnostic performance (“efficacy”) inherently problematic.

In conclusion, S-Patch compared with Holter supported longer continuous monitoring without compromising patient acceptability and was well tolerated in both frail older adults with cognitive impairment. S-Patch may be particularly useful in patients who are unable to tolerate conventional Holter monitoring or where extended monitoring is desirable but invasive approaches are not appropriate.

## Supplementary material

10.2196/81761 Multimedia Appendix 1 Assessment schedule.

10.2196/81761 Multimedia Appendix 2 User preference survey.

10.2196/81761 Multimedia Appendix 3 S-Patch event diary.

10.2196/81761 Multimedia Appendix 4 Comparison of Holter vs S-Patch data.

## Abbreviations

AF: atrial fibrillation
AMT: Abbreviated Mental Test
CFS: Clinical Frailty Scale
CIRB: Centralized Institutional Review Board
ED: emergency department
LOS: length of stay
PPM: permanent pacemaker
SPPB: Short Physical Performance Battery
