# Supplementary material for: Comparing the Feasibility of S-Patch vs 24-Hour Holter in Frail Older People With Falls: Randomized Open-Label Crossover Pilot Study
Source: JMIR Rehabil Assist Technol. 2026 Aug 14;13:e81761. doi: 10.2196/81761 (PMC13475935; doi:10.2196/81761)
Supplement: Supplementary file 1 [file rehab-v13-e81761-s001.docx]

**Supplementary Information 1: Assessment Schedule**

| ***Study Visits*** | ***1^a^*** | ***2*** | ***3*** | ***4*** |
| --- | --- | --- | --- | --- |
| *Window Period** | *- 3 Months to Day -1 Day* | *Day 1* | *Day 2 to Day 29ᵇᶜ* | *Day 5 to Day 30 (+/- 3 days)* |
| *Inclusion / Exclusion criteria* | *X* |  |  |  |
| *Sign informed consent* | *X* |  |  |  |
| *Demographics / Medical history* | *X* |  |  |  |
| *Randomisation* | *X* |  |  |  |
| *Return of allocated device ^b^* |  |  | *X* | *X* |
| *Device Hook up and Monitoring (Either S Patch3-Cardio*/S-Patch Ex *or Holter)* |  | *X* | *X* |  |
| *Issue of Event Diary* |  | *X* | *X* |  |
| *Collection of Event Diary* |  |  | *X* | *X* |
| *Adverse Event Check* |  | *X* | *X* | *X* |
| *Completion of User Preference Survey^d^* |  |  |  | *X* |

^a^ Visit 1 is conducted during subject routine clinical visit

^b^ Either Holter or S Patch3- Cardio/S-Patch will be returned

^c^ Visit 3 will occur between Day 2 to Day 29 should if subject is randomised to either Group A or Group B

^d^ User Preference Survey shall be completed by LAR/Caregiver who is taking care of the subject if the subject is cognitively impaired and consent is obtained from LAR. For inpatient, nurses will complete the survey
