# Supplementary material for: Comparing the Feasibility of S-Patch vs 24-Hour Holter in Frail Older People With Falls: Randomized Open-Label Crossover Pilot Study
Source: JMIR Rehabil Assist Technol. 2026 Aug 14;13:e81761. doi: 10.2196/81761 (PMC13475935; doi:10.2196/81761)
Supplement: Supplementary file 2 [file rehab-v13-e81761-s002.docx]

**Supplementary Information 2:**

# User Preference Survey

**Instruction on Completion:**

Please indicate with a tick 🗹 in the corresponding box.

1. Comparing the Holter and S Patch, which device do you prefer?

Holter	(Answer Q2)

S Patch (Answer Q3)

No preference (Answer Q4)

None of the device (Answer Q5)

2. Why do you prefer Holter?

User friendly and ease of operation

Comfortable

Does not affect daily activities

Others, pls specify: __________________________________________________

3. Why do you prefer the S Patch?

User friendly and ease of operation

Comfortable

Does not affect daily activities

Others, pls specify: __________________________________________________

4. You have indicated that you have no preference over the devices, why is that so?

Both are similar in term of ease of usage

Both are similar in term of comfortable

Both does not affect daily activities

Others, pls specify: __________________________________________________

5. You have indicated that you like neither of the device, why is that so?

Both devices are difficult to use

Both devices are uncomfortable

Others, pls specify: __________________________________________________

6. How satisfied are you with the use of the Holter device?

Extremely satisfied

Very satisfied

Moderately satisfied

Not so satisfied

Dissatisfied

7. How satisfied are you with the use of the S Patch device?

Extremely satisfied

Very satisfied

Moderately satisfied

Not so satisfied

Dissatisfied

Additional Comments if any:

Completed by: _______________________________	Date: ___________________

(Initials)	(dd/mmm/yy)
