# Supplementary material for: Comparing the Feasibility of S-Patch vs 24-Hour Holter in Frail Older People With Falls: Randomized Open-Label Crossover Pilot Study
Source: JMIR Rehabil Assist Technol. 2026 Aug 14;13:e81761. doi: 10.2196/81761 (PMC13475935; doi:10.2196/81761)
Supplement: Supplementary file 3 [file rehab-v13-e81761-s003.docx]

**Supplementary Information 3**

**S Patch Event diary**

**Instruction for use:**

Please use this diary to record down information should you feel giddy or have other symptoms.

Please bring along this event diary during the return of the device.

Start Date of Diary: |___|___|/ |___|___| / 202|___|

End Date of Diary: |___|___|/ |___|___| / 202|___|

| **Time 时间** | **Symptoms 病症** | **Activity活动** |
| --- | --- | --- |
| **e.g. 8pm 晚上八点** | **Heart Beating fast心跳快** | **Watching TV看电视** |
|  |  |  |
|  |  |  |
|  |  |  |
|  |  |  |
|  |  |  |
|  |  |  |
|  |  |  |
|  |  |  |
|  |  |  |
|  |  |  |
|  |  |  |

Diary Completed By: _________________________________ (Initial/ Date)
