# Supplementary material for: Comparing the Feasibility of S-Patch vs 24-Hour Holter in Frail Older People With Falls: Randomized Open-Label Crossover Pilot Study
Source: JMIR Rehabil Assist Technol. 2026 Aug 14;13:e81761. doi: 10.2196/81761 (PMC13475935; doi:10.2196/81761)
Supplement: Supplementary file 4 [file rehab-v13-e81761-s004.docx]

**Supplementary Information 4:**

**Comparison of Holter vs S-Patch data**

| **Variable** | **Holter** | | **S-Patch** | | ***p**** | ***r***** |
| --- | --- | --- | --- | --- | --- | --- |
|  | **SD** | **Mean** | **SD** | **Mean** |  |  |
| APPLICATION TIME (min) |  |  |  |  |  |  |
| RECORDED TIME (hr) |  |  |  |  |  |  |
| TOTAL NUMBER OF BEATS (‘000s) |  |  |  |  |  |  |
| ANALYZED TIME (after removal artefact) |  |  |  |  |  |  |
| ANALYZED TOTAL NUMBER OF BEATS  (after removal artefact) |  |  |  |  |  |  |
| ANALYASIS TIME (min) |  |  |  |  |  |  |
| Minimum heart rate (bpm) |  |  |  |  |  |  |
| Maximum heart rate (bpm) |  |  |  |  |  |  |
| Average heart rate (bpm) |  |  |  |  |  |  |
| R-R interval (s) |  |  |  |  |  |  |
| Single SVE |  |  |  |  |  |  |
| Single VE |  |  |  |  |  |  |
| Total SVE |  |  |  |  |  |  |
| Total VE |  |  |  |  |  |  |
| Atrial fibrillation (%) |  |  |  |  |  |  |
|  | | | | | | |
